# Clinically relevant genomic and phenotypic differences in virulence, antimicrobial resistance, and biofilm-associated tolerance between *Streptococcus suis* lineages ST1 and ST123

**DOI:** 10.1186/s13567-026-01782-2

**Published:** 2026-06-12

**Authors:** Cristina Uruén, Clara M. Marín, Luis Daniel González-Vázquez, Marcelo Gottschalk, Miguel Arenas, Jesús Arenas

**Affiliations:** 1 Unit of Microbiology and Immunology, Faculty of Veterinary, University of Zaragoza, Miguel Servet, 177, 50017 Zaragoza, Spain; 2 Institute Agrofood of Aragón-IA2, University of Zaragoza-CITA, Zaragoza, Spain; 3 Department of Animal Science, Centre of Research and Technology of Aragón (CITA), Zaragoza, Spain; 4 Department of Biochemistry, Genetics and Immunology, University of Vigo, Vigo, Spain; 5 CINBIO, University of Vigo, Vigo, Spain; 6 Research Group On Infectious Diseases in Production Animals and Swine and Poultry Infectious Diseases Research Centre, Faculty of Veterinary Medicine, University of Montreal, Saint-Hyacinthe, QC Canada

**Keywords:** *Streptococcus suis*, virulence, pathogenicity, ST1, ST123, β-lactam resistance, biofilm formation, macrophage killing

## Abstract

*Streptococcus suis* is a Gram-positive bacterium and an important pathogen in pigs and humans. It can be classified into more than 3000 sequence types (STs), among which the ST1 and ST123 lineages are highly prevalent in Spain. ST1 is a globally distributed lineage, while ST123 has emerged within the last decade only in Spain. In this study, we compared the genotypic and phenotypic characteristics of representative isolates from both lineages to better understand the factors driving the emergence of ST123. Comparative genomic analysis revealed higher genetic variability in ST123 than in ST1. The genomes of both lineages share approximately 1429 genes, representing about 61% of the total genome. Among the lineage-specific genes, we identified 131–143 genes encoding proteins involved in diverse biological functions, including metabolism, regulation, transport, and virulence. Some of these genes were located on genetic islands, encoding for proteins involved in nutrition and catabolism of specific carbohydrates. In mouse infection models, both STs showed a strong capacity to cause systemic infection, although they differed in tissue persistence patterns. In macrophage cultures, ST123 isolates showed reduced adherence and intracellular survival compared with ST1. In contrast, ST123 isolates demonstrated the capacity to acquire ampicillin resistance under laboratory conditions. Moreover, ST123 isolates exhibited increased biofilm formation and enhanced tolerance to β-lactam antibiotics within biofilms compared with ST1. In conclusion, the combination of virulence, increased biofilm-associated antibiotic tolerance, and increased  propensity to acquire  antimicrobial resistance may explain the rising prevalence of ST123 in Spain. Its recent detection in Italy further supports its potential for expansion and establishment across Europe in the coming years.

**Supplementary Information:**

The online version contains supplementary material available at 10.1186/s13567-026-01782-2.

## Introduction

*Streptococcus suis*, a Gram-positive bacterium, is a commensal microorganism of the upper respiratory tract and a significant pathogen in pigs. It causes streptococcal disease, characterized by clinical signs such as meningitis, septicemia, arthritis, endocarditis, and pneumonia. This bacterium can be classified into serotypes according to the composition of its capsular polysaccharide. To date, 29 serotypes have been described, with serotypes 2 and 9 being the most frequently recovered from clinically affected pigs in most parts of the world [1]. Based on Multi-Locus Sequence Typing (MLST), *S. suis* can be further classified into over 3000 sequence types (STs). Certain STs are frequently recovered from diseased animals and some of them showed particular genotypic and phenotypic traits [2, 3]. Furthermore, *S. suis* can produce more than 70 putative virulence factors [4, 5]. The presence or absence of some virulence-associated genes has been linked to certain genetic lineages [3], influencing their pathogenicity, tissue tropism, and host interaction [6]. These genetic and phenotypic variations highlight the remarkable heterogeneity within *S. suis*.

Spain is the largest pig producer in Europe, and this industry is steadily growing. We and others have shown that ST1 and ST123 are the most prevalent *S. suis* lineages among clinical isolates recovered from the most relevant pig producing regions in Spain [3, 7]. They are distant lineages, belonging to different clonal complexes (CCs) CC1 and CC123, respectively [3]. ST1 is a globally distributed lineage, frequently associated with severe infections in pigs and humans. The high prevalence of this lineage has been reported in several countries across Europe [2, 3] and Asia [8, 9]. In contrast, ST123 was first identified in clinical isolates from Spain around two decades ago [10], and since then, its prevalence has continued to rise [3, 7]. A recent report identified this ST in Italy [11], suggesting that it is spreading to other European countries. Despite its emergence and international spread, this lineage remains poorly characterized.

While both ST1 and ST123 lineages are highly pathogenic, previous studies suggest important biological and epidemiological differences. First, ST123 and ST1 are phylogenetically unrelated, with their core genomes clustering into distinct genetic groups [3]. Second, ST123 strains produce capsule type 9, whereas ST1 strains produce capsule types 2, 1, 1/2, and 14 [3]. Furthermore, clinical isolates of ST123 are frequently resistant to β-lactam antibiotics, while those of ST1 are often susceptible [12]. These observations suggest that both invasive lineages may use different strategies to establish infection or evade antibiotic treatment. Considering the genetic and phenotypic differences between these STs can be crucial for developing ST-specific control measures and improving therapeutic approaches. In this study, we aimed to characterize and compare the genotypic and phenotypic traits of *S. suis* isolates from ST1 and ST123 recovered in Spain within the same period, with a focus on virulence-associated features and antimicrobial resistance.

## Materials and methods

### Bacterial strains and culture conditions

All *S. suis* strains used in this study are listed in Table 1. A total of 22 *S. suis* isolates were obtained from diseased piglets (3–10 weeks old) across various Spanish Autonomous Communities between 2017 and 2020 [3]. These isolates had been previously characterized for their ST, capsular type, presence of virulence-associated genes, and antimicrobial resistance profiles [3, 12]. Of the 22 isolates, 13 belonged to ST1, and 9 to ST123. While ST1 isolates showed capsules of serotypes 2, (*n* = 3) 1 (*n* = 5), 1/2 (*n* = 3), and 14 (*n* = 2), all ST123 isolates belonged to  serotype 9 (Table 1). For solid culture, bacteria from −80 °C glycerol stocks were plated onto Todd–Hewitt Broth (THB, Oxoid Ltd., Hampshire, UK) supplemented with 1.5% bacteriological agar (THA) and incubated overnight at 37 °C in a jar with a candle. For liquid cultures, single colonies from THA plates were inoculated into THB and incubated statically at 37 °C for approximately 18 h. To simulate in vitro growth under stress conditions, previous methods were applied [13, 14] with minor modifications. Briefly, single colonies from overnight THA cultures were propagated into THB medium at an optical density of 600 nm (OD_600_) of 0.2. Then, 10 µL of this bacterial suspension was added to 90 µL of THB, with and without supplementation with 2 mM hydrogen peroxide (2 mM H_2_O_2;_ PanReac, Barcelona, Spain) in 96-well flat-bottom microplates (Corning, Kennebunk, USA), and incubated in a CLARIOstar microplate reader (BMG Labtech, Cary NC). The OD_600_ was measured every 30 min. Each assay was performed at least in triplicate (three independent days)**.** For comparison of bacterial growth curves, the area under the curve (AUC) was calculated from three independent experiments. Statistical significance between treated and untreated groups was determined using an unpaired Student´s *t*-test performed in GraphPad Prism (v8.0.1, Dotmatics), with *p*-values lower  than 0.05 considered significant. The same method was used to evaluate bacterial growth in the presence of ampicillin at concentrations of 0, 0.015, 0.12, and 1 mg/L.

**Table 1:** **Bacterial strains used in the study**

Isolate	Relevant characteristics	Serotype; CC	Isolation date (month/year)	MIC AMP (mg/L)	Sensitivity^1^ to AMP	Accession number	Ref.
*Lineage ST1*							
Ss_02	Isolated from joints in Aragón	14; 1	02/2019	0.06	S	NZ_JAWWZM010000000	[3]
Ss_02_R1_0.12	Spontaneous AMP-resistant obtained from Ss_02	14; 1		0.12	S		This study
Ss_02_R2_0.12	Spontaneous AMP-resistant obtained from Ss_02	14; 1		0.12	S		This study
Ss_02_R3_0.12	Spontaneous AMP-resistant obtained from Ss_02	14; 1		0.12	S		This study
Ss_02_R4_0.12	Spontaneous AMP-resistant obtained from Ss_02	14; 1		0.12	S		This study
Ss_02_R5_0.12	Spontaneous AMP-resistant obtained from Ss_02	14; 1		0.12	S		This study
Ss_21	Isolated from joints in Navarra	1/2; 1	03/2020	≤ 0.06	S	NZ_CP139881	[3]
Ss_22	Isolated from joints in País Vasco	14; 1	02/2019	0.06	S	NZ_CP139880	[3]
Ss_22_R1_0.12	Spontaneous AMP-resistant obtained from Ss_22	14; 1		0.12	S		This study
Ss_22_R2_0.12	Spontaneous AMP-resistant obtained from Ss_22	14; 1		0.12	S		This study
Ss_22_R3_0.12	Spontaneous AMP-resistant obtained from Ss_22	14; 1		0.12	S		This study
Ss_22_R4_0.12	Spontaneous AMP-resistant obtained from Ss_22	14; 1		0.12	S		This study
Ss_22_R5_0.12	Spontaneous AMP-resistant obtained from Ss_22	14; 1		0.12	S		This study
Ss_27	Isolated from CNS in Castilla La-Mancha	1; 1	10/2019	≤ 0.06	S	Submitted	[3]
Ss_31	Isolated from joints in Cataluña	1; 1	10/2018	0.06	S	Submitted	[3]
Ss_64	Isolated from joints in Galicia	1; 1	03/2018	< 0.06	S	Submitted	[3]
Ss_72	Isolated from CNS in Madrid	2; 1	06/2019	< 0.06	S	NZ_JAXKWL000000000	[3]
Ss_80	Isolated from joints in Castilla La-Mancha	1; 1	01/2018	0.06	S	NZ_JAWWZJ010000000	[3]
Ss_81	Isolated from joints in Cataluña	1/2; 1	08/2017	< 0.06	S	Submitted	[3]
Ss_105	Isolated from joints in Castilla y León	1/2; 1	08/2020	< 0.06	S	Submitted	[3]
Ss_121	Isolated from CNS in Aragón	1; 1	02/2019	< 0.06	S	NZ_JAWWZF010000000	[3]
Ss_151	Isolated from joints in Navarra	2; 1	11/2018	0.06	S	Submitted	[3]
Ss_165	Isolated from CNS in Aragón	2; 1	11/2019	< 0.06	S	Submitted	[3]
*Lineage ST123*							
Ss_50	Isolated from joints in Galicia	9; 123	10/2018	< 0.06	S	Submitted	[3]
Ss_84	Isolated from CNS in Cataluña	9; 123	01/2019	0.25	S	NZ_JAXKWK010000000	[3]
Ss_84_R1_0.5	Spontaneous AMP-resistant obtained from Ss_84	9; 123		0.5	I		This study
Ss_84_R2_0.5	Spontaneous AMP-resistant obtained from Ss_84	9; 123		0.5	I		This study
Ss_84_R3_0.5	Spontaneous AMP-resistant obtained from Ss_84	9; 123		0.5	I		This study
Ss_84_R4_0.5	Spontaneous AMP-resistant obtained from Ss_84	9; 123		0.5	I		This study
Ss_84_R5_0.5	Spontaneous AMP-resistant obtained from Ss_84	9; 123		0.5	I		This study
Ss_84_R1_2	Spontaneous AMP-resistant obtained from Ss_84	9; 123		4	R		This study
Ss_84_R2_2	Spontaneous AMP-resistant obtained from Ss_84	9; 123		4	R		This study
Ss_84_R3_2	Spontaneous AMP-resistant obtained from Ss_84	9; 123		4	R		This study
Ss_84_R4_2	Spontaneous AMP-resistant obtained from Ss_84	9; 123		4	R		This study
Ss_84_R5_2	Spontaneous AMP-resistant obtained from Ss_84	9; 123		4	R		This study
Ss_100	Isolated from CNS in Aragón	9; 123	03/2019	0.5	I	NZ_JAWWZG010000000	[3]
Ss_106	Isolated from CNS in Castilla y León	9; 123	10/2018	0.5	I	NZ_JAXKWJ010000000	[3]
Ss_110	Isolated from CNS in Andalucía	9; 123	01/2019	0.12	S	Submitted	[3]
Ss_146	Isolated from CNS in Castilla y León	9; 123	10/2019	0.25	S	Submitted	[3]
Ss_150	Isolated from joints in Murcia	9; 123	10/2019	0.12	S	Submitted	[3]
Ss_156	Isolated from CNS in Aragón	9; 123	03/2019	0.12	S	NZ_JAWWZE010000000	[3]
Ss_156_R1_0.5	Spontaneous AMP-resistant obtained from Ss_156	9; 123		0.5	I		This study
Ss_156_R2_0.5	Spontaneous AMP-resistant obtained from Ss_156	9; 123		0.5	I		This study
Ss_156_R3_0.5	Spontaneous AMP-resistant obtained from Ss_156	9; 123		0.5	I		This study
Ss_156_R4_0.5	Spontaneous AMP-resistant obtained from Ss_156	9; 123		0.5	I		This study
Ss_156_R5_0.5	Spontaneous AMP-resistant obtained from Ss_156	9; 123		0.5	I		This study
Ss_156_R1_2	Spontaneous AMP-resistant obtained from Ss_156	9; 123		2	I		This study
Ss_156_R2_2	Spontaneous AMP-resistant obtained from Ss_156	9; 123		2	I		This study
Ss_156_R3_2	Spontaneous AMP-resistant obtained from Ss_156	9; 123		2	I		This study
Ss_156_R4_2	Spontaneous AMP-resistant obtained from Ss_156	9; 123		2	I		This study
Ss_156_R5_2	Spontaneous AMP-resistant obtained from Ss_156	9; 123		2	I		This study
Ss_160	Isolated from CNS in Aragón	9; 123	06/2019	0.12	S	Submitted	[3]

^1^The antibiotic susceptibility phenotype of each isolate, based on EUCAST-established breakpoints, is indicated as sensitive (S), intermediate (I), or resistant (R)

ST: sequence type; CC: clonal complex. AMP: ampicillin. MIC: minimal inhibitory concentration

### DNA extraction and whole genome sequencing

Chromosomal DNA from *S. suis* isolates Ss_151 (ST1) and Ss_150 (ST123) was extracted from bacterial pellets harvested from fresh THB cultures grown until the exponential phase using the Wizard® Genomic DNA Purification Kit (Promega, USA). The extracted genomic DNA was sequenced using the Illumina HiSeq 2500 platform at STAB Vida Lda (Caparica, Portugal). DNA-seq libraries of the *S. suis* isolates were assembled following previously described protocols [3, 12].

### Comparative and evolutionary genomic analyses

A total of 22 *S. suis* genomes were used for comparative genomic analysis (Table 1). Of these, two genomes were newly sequenced in this study (see above), while the remaining 20 were previously published by us [3, 12] or are part of a manuscript currently under review. For pangenome analysis, the Roary pipeline was used [15]. The protein sequences of selected genes were analyzed using BLASTp searches against the NCBI database, and their putative functions were predicted on the basis of sequence homology. Nucleotide diversity (π) was calculated with the Pegas package in R [16], which allowed to estimate intra-lineage genetic variation. To compare the distribution of nucleotide frequencies along sequences between the ST datasets, the Kullback–Leibler divergence was used [17], following previous studies [18]. Next, the rates of molecular evolution for both datasets were estimated for which the best-fitting substitution model of DNA evolution for each dataset was identified with ModelTest-NG [19], based on the Bayesian Information Criterion according to a previous study [20]. Next, we inferred a maximum-likelihood phylogenetic tree with *RAxML-NG* [21] under the GTR + G + I substitution model, which was previously selected, with default settings. Subsequently, the rate of evolution was estimated using *BEAST 2* (version 2.6.7) [22], under the previously selected substitution model of molecular evolution and a strict molecular clock model. The clock model yielded likelihood estimates similar to those of relaxed models with more parameters and was therefore statistically preferred. We applied a sufficient number of MCMC iterations to ensure convergence, as indicated by an effective sample size greater than 200 for the estimates of the rate of evolution, according to [22] and assessed using Tracer [23].

### Ancestral sequences of proteins involved in β-lactam resistance

We investigated the evolutionary divergence between ST1 and ST123 proteins involved in β-lactam resistance, through the fixation of amino acid changes over time, using ancestral sequence reconstruction. In particular, the sequences of PBP1A, PBP2B, and PBP2X proteins obtained from ST1 and ST123 isolates were analyzed. The best-fitting empirical substitution model of protein evolution was identified with *ModelTest-NG* considering the Bayesian Information Criterion. Next, a maximum-likelihood phylogenetic tree, based on the previously selected substitution model, was inferred with *RAxML-NG* [21]. Finally, the sequences of the ancestral nodes of the reconstructed phylogenetic tree were inferred with *RAxML-NG* through a maximum-likelihood phylogenetic tree, considering the previously reconstructed phylogenetic tree and the selected substitution model of protein evolution.

### Animal experiments

The animal experiments performed in this study were approved by the Ethical Committee for scientific procedures on Animals of the Centro de Investigación y Tecnología Agroalimentario de Aragón (CITA), according to the Spanish legislation on animal experimentation (permit number CITA-2023–01). Animals were obtained from Janvier-labs, a  company compromised with animal welfare and expert in rodent research models. The 4-week-old CD1 mice were randomly assigned to groups of six animals. After a 7-day acclimatization period, mice were infected intranasally following the protocol previously described [24]. Shortly, mice were anesthetized with isoflurane (IsoFlo, Zoetis Spain S.L.), and 12.5 µL of 1% acetic acid was added into each nostril. After 1 h, animals were reanesthetized, and each group was infected with 3–8 × 10^8^ colony-forming units (CFU) of a *S. suis* strain. The infection was performed with five isolates of ST1 (Ss_02, Ss_22, Ss_31, Ss_80, and Ss_151) and five isolates of ST123 (Ss_84, Ss_100, Ss_106, Ss_110, and Ss_156), all of them tetracycline-resistant strains [12]. After 3 and 7 days post-infection (dpi), animals were euthanized by cervical dislocation. Organs, including the spleen, brain, lungs, heart, joints, and nostrils were aseptically collected, weighed, diluted in phosphate buffer saline (PBS; Na_2_HPO_4_ 7.7 mM, Na_2_HPO_4_ 2.6 mM, NaCl 145.5 mM, pH 7.2), and homogenized using a stomacher. Organ homogenates were serially diluted and plated onto THA supplemented with 10 mg/L of tetracycline for selective enumeration of CFUs. The Mann–Whitney *U* signed-rank test was applied for statistical comparison using GraphPad Prism.

### Eukaryotic cell cultures

The J774A.1 murine macrophage cell line was cultured in Dulbecco’s modified eagle medium supplemented with 5% non-heat-inactivated fetal calf serum at 37 °C in a humidified atmosphere with 5% CO_2_. All cell culture reagents were obtained from Thermo Fisher Scientific (Waltham, MA, USA). Cells were maintained in 25 cm^2^ tissue culture flasks without antibiotics and passaged when they reached approximately 80% confluence. For infection assays, cells between passages 4 and 25 were seeded in 24-well plates 2 days prior to the experiment.

### Adherence and intracellular survival

The adherence to and intracellular survival of *S. suis* in J774A.1 murine macrophages were assessed as previously described [25, 26]. Briefly, prior to infection, macrophages were stimulated with 1 µg/mL of *Escherichia coli* lipopolysaccharide (LPS, Sigma-Aldrich, Darmstadt, Germany) for 24 h. Cells were then infected with *S. suis* at a multiplicity of infection of 100. To enhance bacterium–macrophage contact, plates were centrifuged at 120 × *g* for 5 min. After 1.5 h, nonadherent bacteria were removed by sequential washings with Dulbecco’s PBS (Thermo Fisher Scientific, DE, USA). Viable adherent bacteria were quantified by lysing the macrophage monolayer with 1% saponin in Dulbecco’s PBS for 15 min, followed by mechanical disruption. The number of CFUs was determined by plating serial dilutions onto THA. For intracellular survival assays, after 1.5 h of infection, nonadherent bacteria were removed by washing, and remaining extracellular bacteria were inactivated by treatment with 120 µg/mL of gentamicin for 1 h. Gentamicin was then removed by washing, and viable intracellular bacteria were quantified as described above. In parallel, cell-associated and extracellular bacteria were evaluated in each experiment to assess the level of bacterial interaction with macrophage monolayers and their proliferation in the culture medium. Results are expressed as the percentage of cell-associated and gentamicin-resistant (internalized) bacteria relative to the initial inoculum. Statistical analyses were performed using an unpaired *t* test with GraphPad Prism, with *p* value lower  than 0.05 considered statistically significant.

### Adaptive resistance assays and determination of minimum inhibitory concentration (MIC)

An adapted version of the protocol described by Nishimoto, et al. [27] was used to evaluate the in vitro development of ampicillin resistance. To do so, an overnight culture of *S. suis* grown in THB was used to inoculate fresh THB medium supplemented with ampicillin at an initial concentration of 0.002 mg/L, which was the concentration at which most sensitive ST1 strains could grow in a similar manner to their wildtype. Bacteria were serially passaged in THB with progressively increasing ampicillin concentrations, increased 1.5 fold  at each  passage (up to a maximum of 2 mg/L). Every 12 h, grown cultures showing an OD_600_ higher than 0.3 were collected by centrifugation (5000 × *g* for 10 min), bacterial pellets were then resuspended in pre-warmed fresh medium adjusted to an initial OD_600_ of 0.1 and incubated again at 37 °C in a humidified 5% CO_2_ atmosphere. Cultures were discontinued when the OD_600_ failed to reach 0.3 after 12 h of incubation. The MIC to ampicillin was determined using the broth microdilution method [12] considering the clinical breakpoints established by the European Committee on Antimicrobial Susceptibility Testing (EUCAST) [28].

### Amplification and sequencing of the pbp2x gene

The *pbp2x* gene sequence was obtained by PCR using the primers pbp2x_Seq (5′-TTATGCAAACGCGGCTTTT-3′) and mraY_Rev (5′-CGTTTGAATAACCACTCCATAT-3′’). For isolate Ss_84, the primer pair pbp2x-mraY_EXT_Fw (5′-AGAGATTGATGGGATTTGTTATGATTT-3′) and pbp2x-mraY_EXT_Rev (5′-CGACAAATTCGCCATTTTTCATTT-3′) was used as previously described  [12]. PCR reactions were performed in a final volume of 50 µL, with 0.32 µM of each primer, 240 µM of dNTPs, 2.5 mM MgCl_2_, 0.1 U/µL of NZYTaq II DNA polymerase (NZYtech, Lisbon, Portugal) and reaction buffer. Thermal cycling conditions were 94 °C for 5 min for initial denaturation, 30 cycles of 94 °C for 20 s, 55 °C for 30 s, and 68 °C for 5.5 min, and a final extension at 68 °C for 7 min. PCR products were visualized on a 1% agarose gel stained with Green® Nucleic Acid Stain (Sigma-Aldrich, Darmstadt, Germany) using an iBright Imaging System (Thermo Fisher Scientific, DE, USA). Bands of interest were purified using the PCR Product Pre-Sequencing Kit (Thermo Fisher Scientfic, DE, USA), following the manufacturer´s instructions. Purified amplicons were sequenced by the Sanger method at StabVida (Caparica, Portugal). The obtained DNA sequences were translated, and the resulting amino acid sequences were inspected to identify potential spontaneous mutations.

### Biofilm formation and biofilm tolerance to ampicillin

Biofilm formation capacity was assessed according to our previously described protocol [29]. Briefly, a 10–12-h culture of *S. suis* grown in THB was used to inoculate fresh THB to an initial OD_600_ of 0.05. Cultures were incubated at 37 °C in a humidified 5% CO_2_ atmosphere until reaching an OD_600_ of 0.2. Then, 100 µL of the bacterial suspension was transferred to the wells of a 96-well polystyrene tissue culture plate (Corning Incorporated, Kennebunk, ME, USA) and incubated for 24 and 48 h under the same conditions. After incubation, the supernatant was carefully removed, and the wells were gently washed with distilled water (dH_2_O) to eliminate nonadherent cells. Biofilms were stained by adding 100 µL of 0.5% violet crystal to each well and incubating for 2 min. Wells were then washed with sterile dH_2_O. Adherent biofilms were solubilized by adding 100 µL of 33.3% acetic acid, and biofilm biomass was quantified by measuring the optical density at 595 nm using a microplate spectrophotometer (Sunrise, Tecan).

To assess ampicillin tolerance in biofilms, 24-h biofilms were first established in 96-well plates by incubating cultures at 37 °C in a 5% CO_2_ atmosphere. Following incubation, the supernatant was carefully removed, and the wells were gently washed with sterile dH_2_O to remove nonadherent cells. Fresh THB supplemented with ampicillin was added to each well, and plates were incubated for an additional 24 h under the same conditions. After treatment, the supernatant was discarded, and wells were gently washed with sterile dH_2_O. Biofilms were resuspended in 100 µL of fresh THB, serially diluted tenfold, and plated onto THA plates. Plates were incubated for 24 h at 37 °C with 5% CO_2_. CFU were counted and compared with those from untreated controls (THB without ampicillin) to evaluate the relative tolerance of biofilm-embedded bacteria.

## Results and discussion

### Genomic comparative analysis of ST1 and ST123

A panel of 22 genomes from invasive *S. suis* isolates of ST1 (*n* = 13) and ST123 (*n* = 9) lineages, obtained from geographically distant locations in Spain, was analyzed for comparative genomics. The observed nucleotide diversity (π) in ST1 and ST123 isolates was 0.13% and 0.16%, respectively. When both populations were analyzed together, π increased to 1.34%, indicating substantial genetic divergence between the two groups. The Kullback–Leibler divergence between ST1 and ST123 genome data, which reflects the distance between these STs in terms of distribution of nucleotide frequencies along their sequences, was 4.31%, a value considerably high for members of the same species. The estimated rate of evolution was 1.51 × 10^–5^ (9.90 × 10^–6^–2.11 × 10^–5^, 95% HDPI) and 8.34 × 10^–6^ (6.19 × 10^–6^–1.07 × 10^–5^, 95% HDPI) substitutions per site per year for ST123 and ST1, respectively, which indicate that ST123 accumulates observed genetic changes at a higher, but not significant, rate than ST1.

**Figure 1:**
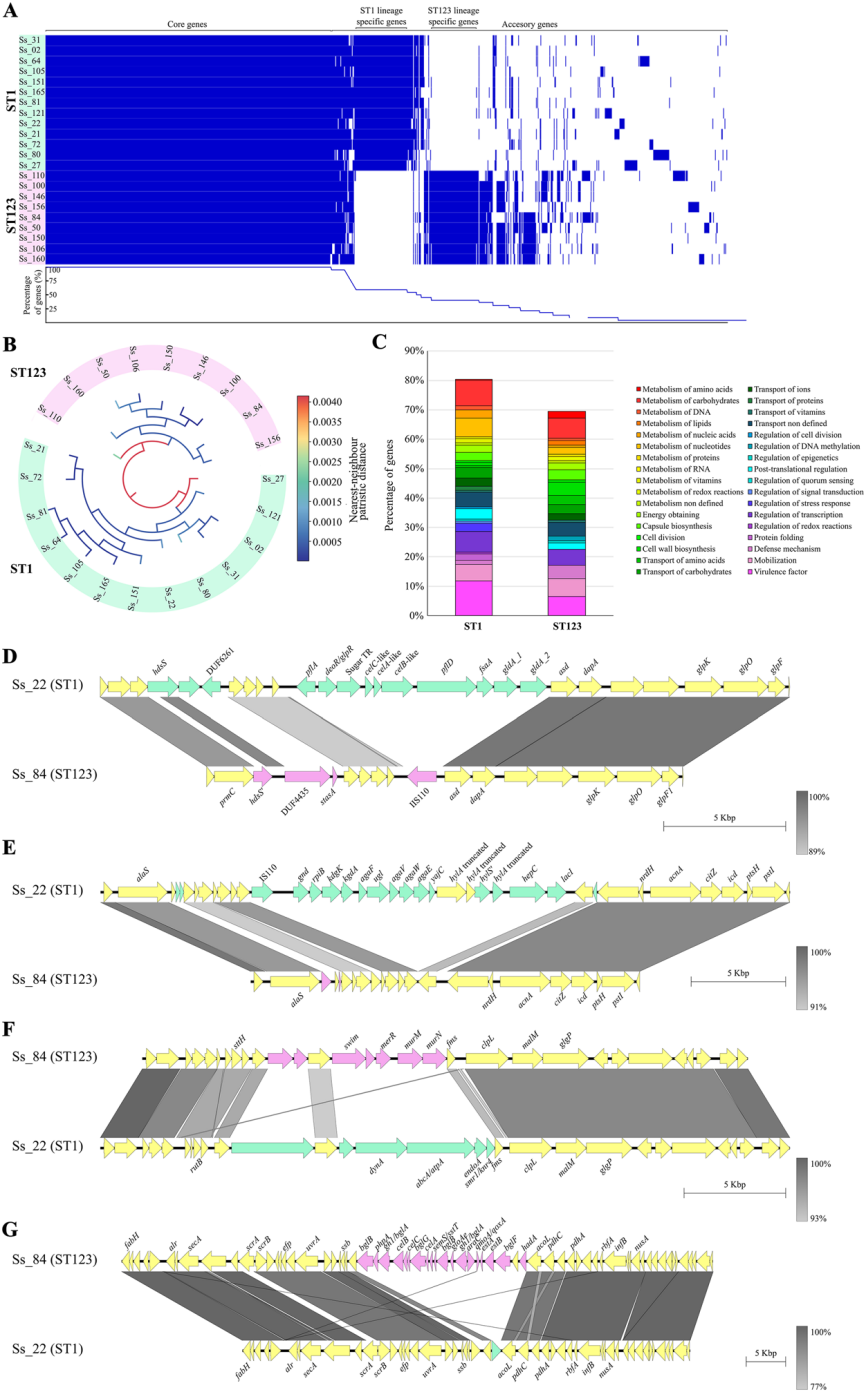
**Comparative genomic and genetic analysis of ST1 and ST123 isolates.** **A** Pangenomic analysis of 13 ST1 and 9 ST123 genomes represented in a matrix of gene content. Each row corresponds to an isolate. Gene presence is indicated in blue and absence in white. The percentage of isolates containing each gene is shown below the matrix. **B** Maximum-likelihood phylogenetic tree inferred with *RAxML-NG* from the core-genome alignment of ST1 and ST123 under the GTR + G + I substitution model of evolution (which was the best-fitting likelihood-based model, see Methods). Branch lengths are shown in different  colors (adapted to nearest-neighbor patristic distance) as indicated in the legend. **C** Biological categorization of lineage-specific genes for each ST. Genes with unknown functions were excluded. **D**–**G** Comparison of the genomic contexts of genomic islands identified in representative *S. suis* Ss_22 (ST1) and Ss_84 (ST123). ST1-specific genes are colored in pink, and ST123-specific genes in green, while common genes are colored in yellow. Shared regions and their sequence identities are indicated in gray scale, as specified in each panel.

Pangenome analysis identified 2491 genes in ST1 and 2561 genes in ST123, with 1743 genes shared between both lineages. The combined pangenome comprised 3420 genes, of which 1429 were common to isolates from both STs (Figure 1A). Phylogenetic analysis of the core genome revealed that ST1 and ST123 isolates clustered according to their respective lineages, with three major subclusters in ST1, showing a patristic distance generally lower than that  observed in ST123 isolates (which displayed three major subclusters) (Figure 1B). Among the nonshared genes (accessory genome), a group of lineage-specific genes was identified, comprising 143 genes present in all ST1 isolates and 131 genes in ST123 isolates (Additional file 1). The remaining genes were variably distributed among lineages. A functional classification of lineage-specific genes was performed by comparing all predicted protein sequences against public databases. Genes were grouped into seven major functional categories: (1) metabolism, (2) regulation, (3) transport, (4) mobilization, (5) virulence factors, (6) defense mechanism, and (7) protein folding, and further categorized into specific functions (Figure 1C). In both genotypes, the most represented category among lineage-specific genes was metabolism-related functions (35 and 31 genes for ST1 and ST123, respectively), followed by regulation (23 genes in ST1 and 13 genes in ST123) and transport (22 genes in ST1 and 20 genes in ST123). Particularly, ST1 genomes were enriched in genes encoding regulators (23 genes, compared with 13 in ST123) and virulence factors (22 genes, compared with 13 in ST123), whereas ST123 genomes were enriched in genes encoding proteins involved in defense mechanisms (6 genes, compared with 2 genes in ST1). Genes encoding proteins involved in protein folding were detected only in ST1 isolates. Both lineages carried eight mobilization-related genes, most of which encoded transposases belonging to different families. Examples of genes from each  category are indicated in Table 2. Interestingly, several genes were located within genomic islands containing insertion sequences (Figure 1D, E), suggesting mobilization of these regions. Comparative genomics between both STs revealed the replacement of certain chromosomal segments (Figure 1F), while other islands were inserted within genes or short intergenic regions (Figure 1D, E, G), indicating acquisition or loss of genetic material.

**Table 2:** **Representative lineage-specific genes of ST1 and ST123**

Gene name	Gene product	Gene name	Gene product
*Lineage ST1*		*Lineage ST123*	
*Metabolism*			
*gldA*	Glycerol dehydrogenase	*bglX’*	Truncated beta-glucosidase
*fsA*	Fructose-6-phosphate aldolase	*glf*	Galactose metabolism
*kdgK*	2-keto-3-deoxygluconate kinase	*murM*	Cell wall biosynthesis
*kgdA*	2-keto-3-deoxy-6-phosphogluconate aldolase	*murN*	Cell wall biosynthesis
*nudF*	ADP-ribose pyrophosphatase	*smi1*/*knr4*	SMI1/KNR4 family protein
*pflD*	Pyruvate formate-lyase D	*bglB*	6-phospho-beta-glucosidase
*pflA*	Pyruvate formate-lyase activating protein		
*pdxK*	Pyridoxamine kinase		
*Transport*			
*agaF*	PTS system N-acetylgalactosamine-specific IIB component	*tcyA*	L-cystine transport substrate-binding protein
*agaV*	PTS system N-acetylgalactosamine-specific IIC component	*tcyB*	L-cystine transport permease
*agaW*	PTS system N-acetylgalactosamine-specific IIB component	*tcyC*	L-cystine ABC ATP-binding protein
*agaE*	PTS system N-acetylgalactosamine-specific IIA component	*celA*	Cellobiose PTS system subunit IIB
*celB*-like	PTS sugar transporter IIC component	*celB*	Cellobiose PTS system subunit IIC
*celA*-like	PTS sugar transporter subunit IIB	*celC*	Cellobiose PTS system subunit IIA
*celC*-like	PTS lactose/cellobiose transporter subunit IIA	*bglF*	beta-glucoside-specific PTS system
*ssuA*	Sulfonate transport system substrate-binding protein		
*yajC*	Preprotein translocase subunit YajC		
*pulG*	Type II secretory pathway, pseudopilin PulG		
*pnuC*	Nicotinamide riboside transporter PnuC		
*Regulation*			
*deoR/glpR*	DeoR/GlpR family DNA-binding transcription regulator	*licT*	BglG family transcriptional regulator
*revS*	Two-component regulator	*araC*	AraC-type transcriptional regulator
*relB*	Toxin of toxin-Antitoxin system	*merR*	MerR-family transcriptional regulator
*relE*	Antitoxin of toxin-antitoxin system		
*comX*	Competence regulator		
*Virulence-associated factors*			
*hylS’*	Truncated hyaluronidase	*cps9E*	Nucleoside-diphosphate sugar epimerase
*hepC*	Heparinase II/III family protein	*cps9F*	Capsular biosynthesis protein
*sbp2’*	Truncated major pilus subunit protein	*cps9G*	Glycosyltransferase family 2 protein
*cps2K*	Capsular biosynthesis glycosyltransferase	*cps9H*	Glycosyltransferase
*cps2L*	Acyltransferase family protein	*cps9I*	Glycosyltransferase
*cps2N*	Glycosyltransferase family protein	*cps9J*	Capsular polysaccharide synthesis protein
*cps2O*	Lipopolysaccharide biosynthesis protein	*cps9L*	2-C-methyl-D-erythritol 4-phosphate cytidylyltransferase
*neuB/cps2P*	N-acetylneuraminate synthase	*cps9M*	Capsular polysaccharide biosynthesis
*cps2Q*	UDP-N-acetylglucosamine 2-epimerase	*cps9N*	NAD-dependent epimerase/dehydratase family protein
*neuC/cps2R*	UDP-N-acetylglucosamine 2-epimerase	UN722_00706	Putative fibronectin-binding protein
*neuA/cps2S*	Acylneuraminate cytidylyltransferase	UN722_01091	LPXTG cell wall anchor domain-containing protein
*spa*	SpaA isopeptide-forming pilin-related protein		
*sfp1*	LPXTG-anchored major pilin		

### Lineage-specific genes involved in metabolism and transport

Among metabolism-related genes specific to ST1 isolates, 11 were linked to carbohydrate metabolism, 8 to nucleotide metabolism, 4 to nucleic acid metabolism, and 3 to energy production. Genes involved in carbohydrate metabolism included four involved in the glucuronic acid pathway and others associated with glycerol, galactose, gluconate. and tagatose metabolism. Examples are listed in Table 2. Notably, *gldA* (glycerol dehydrogenase) and *fsA* (fructose-6-phosphate aldolase) form part of an island in all ST1 isolates (Figure 1D). Moreover, this genomic island was also identified by BLAST analysis in several *S. suis* genomes of ST1, showing 100% coverage and 99.9% sequence identity. Some of the genomes of strains with worldwide distribution include P1/7 (AM946016.1), S10 (LR738721.1), SC84 (FM252031.1), ISU1606 (CP030017.1), YP20190405 (CP065431.1), strain 10 (CP058742.1), M104300_S20 (CP102137.1), ZY05719 (CP007497.1), and cnzyss2-311 (CP139163.1), among others. These strains belong to ST1 and ST7, both lineages belong to CC1. These enzymes convert glycerol to fructose-6-phosphate, enabling its use as a carbon source and contributing to NADH/NAD^+^ balance under anaerobic conditions. Both genes likely form an operon with *pflA* and *pflD*, which encode pyruvate formate-lyases activating enzyme that induces mixed-acid fermentation under anaerobic conditions. This pathway function under anaerobic conditions, converting pyruvate into acetyl-CoA and formate via pyruvate formate-lyase (PflB), and producing ATP through acetate formation (PTa/AckA). Under aerobic and microaerophilic conditions, *S. suis* produces lactate via homolactic fermentation, an efficient process that depends on a balanced NAD^+^/NADH ratio. Under anaerobic conditions, limited NAD^+^ regeneration slows glycolysis and decreases energy yield. The mixed-acid fermentation pathway bypasses this redox constraint, providing an alternative energy source in the absence of oxygen and enabling bacterial adaptation to low-oxygen niches such as biofilms or host tissues during infection. While both STs contain genes of the fermentation pathway, *pflA* and *pflD* occur only in ST1, suggesting differences in pathway activation. The presence of *pflA*, *pflD, gldA* and *fsaA* in the same island may facilitate coordinated regulation of anaerobic pyruvate and sugar metabolism. Indeed, upstream regulatory genes encoding LacR and DeoR-type regulators are present, and a putative sugar transporter (*celA*/*C*-like) maybe dedicated to introducing the substrates into the cell. Studies in *Streptococcus pneumoniae* have shown that PflB influences cell membrane lipid composition and contributes to virulence across different tissues [30], while recent work in *S. suis* revealed that PflB regulates bacterial morphology, stress tolerance, and capsular polysaccharide production, and is associated with its capacity to cause bacteremia [31]. ST1 genomes also include specific genes for nucleotide metabolism, two linked to purine and two to pyrimidine metabolism (Table 2). Among them, *nudF* encodes an ADP-ribose pyrophosphatase that hydrolyzes various dinucleotides (ADP-ribose, NADH, NADPH, 8-oxo-dGTP), maintaining the NAD^+^/NADH balance, removing oxidized nucleotides, and controlling intracellular ADP-ribose. This may enhance redox homeostasis and protection against oxidative stress during infection.

A distinct genomic island carries *kdgK* and *kdgA*, genes of the glucuronic acid pathway enabling utilization of uronic acids such as glucuronate or galacturonate from the host (Figure 1E). Although the required *uxaC*, *uxuB*, and *uxuA* genes are absent, the island includes a gene that encodes for a heparinase that degrades heparin and heparan sulfate to release glucuronic acid, and an operon encoding an N-acetyl-galactosamine PTS transporter (*agaF, agaV, agaW, agaE*). Thus, *kdgK* and *kdgA* may contribute to the degradation of glucuronate or gluconate derivatives derived from host mucins and imported through this transporter. Upstream of *kdgK* are *gnd* and *rpiB* genes that encode enzymes of the pentose phosphate pathway, possibly facilitating conversion of these intermediates.

Additional ST1-specific genes encode diverse transport systems, including seven PTS components for carbohydrate uptake, seven ABC transporters (three of them likely involved in ion transport), two multidrug efflux proteins, one CLC family ion exchanger, one major facilitator permease, one amino acid transporter, and proteins YajC and PulG-like related to secretion. The *pnuC* gene encodes a nicotinamide mononucleotide riboside transporter for NAD^+^ precursor uptake, therefore playing a role in cellular energy metabolism, DNA repair, and redox balance. It is clustered with ST1-specific genes, including a MutT/NUDIX hydrolase that hydrolyzes oxidized nucleotides. ST1 also harbors vitamin B_3_ and B_6_ transporters absent in ST123.

Among metabolic genes specific to the ST123 lineage, seven were related to carbohydrate metabolism, six to cell wall biosynthesis, three to nucleotide metabolism, three to amino acids metabolism, three to energy production, two to lipid metabolism, two to redox reactions, and one each to other processes (cell division, DNA, nucleic acids, and protein metabolism). Of those involved in carbohydrate metabolism, four participated in starch/sucrose metabolism and glycolysis/gluconeogenesis pathways, and one in galactose metabolism (Table 2). In ST123 isolates, a truncated *bglX* encodes a 648-amino-acid β-glucosidase instead of the 800-amino-acid enzyme in ST1 isolates. In Gram negative bacteria, such as *Pseudomonas aeruginosa* [32] and *E. coli* [32, 33], BglX hydrolyses β-glucosides to release glucose. Because its C-terminal region contains binding motifs essential for stability and substrate recognition, truncation in ST123 likely reduces or abolishes activity. Disruption of *bglX* in *P. aeruginosa* reduced biofilm formation [32], but strain dependent effects have been described in *E. coli* [34].

A metabolic island in ST123 contains two catabolic modules for β-glucosides (Figure 1G). The first is an operon encoding a PTS transport for cellobiose/β-glycosides uptake (*celB, celC, celA*), a glycoside hydrolase, and a histidine phosphatase. *celA* encodes a phospho-β-glucosidase that transfers phosphate from HPr to CelB and CelC. Deletion of *celA* in *S. mutans* abolishes growth on cellobiose [35], and *celA* overexpression during competence may facilitate nutrient acquisition [36]. Although ST1 genomes encode a predicted cellobiose-like PTS, amino acid identity with the ST123 transporter is < 40% for subunits IIC/IIA. Utilization of carbohydrates such as sucrose or cellobiose has been linked to colonization and virulence in streptococci [37, 38], suggesting that these transporters may differentially influence persistence between STs. The second module encodes a PTS for aryl-β-glucosides (salicin, arbutin, esculin), a hydrolase producing glucose-6-phosphate and aglycones, a quinol monooxygenase, a carboxyl esterase detoxifying phenolic aglycones, and a small sugar transporter, likely regulated by an AraC-type protein. Together, these modules enable ST123 to use neutral and aromatic β-glucosides from plant material in feed. Because aromatic glucosides can occur in the pig oral cavity and cellulose is degraded to cellobiose by gut microbiota, this capacity may promote ST123 persistence in the intestine and possibly systemic invasion through the gut [39].

ST123 genomes also carry two *glf* copies, encoding UDP-galactopyranose mutase, which converts UDP-galactopyranose to UDP-galactofuranose—a precursor of the capsule and cell wall (discussed later). The second *glf* copy, adjacent to the capsule locus, has been proposed as a serotype 9 capsule marker [40] and is associated with virulence through protection from host immunity. Additional glycolytic genes in ST123 participate in reactions converting pyruvate to S-acetyl-dihydrolipoamide-E and salicin/arbutin phosphates to β-D-glucose-6-phosphate, although isoenzymes are shared with ST1. Furthermore, the *murM* and *murN* genes, which encode enzymes involved in peptidoglycan metabolism and cell wall synthesis, were detected exclusively in ST123 genomes (Figure 1F). Their expression enhances crosslinking and has been associated with penicillin resistance in *S. pneumoniae* [41]. The presence of these genes may partly explain the greater β-lactam resistance observed in ST123 clinical isolates compared with ST1 [11, 12]. In contrast, the equivalent ST1 region contains genes encoding membrane-maintenance and cell wall-stability proteins (dynamin-like, ATP-binding, and SMI1/KNR4 family proteins).

Among ST123 lineage-specific transporters, ten genes encoding ABC transporter component were identified, three likely involved in amino acid uptake, one in ion transport, and six with unknown substrates. Homologs of *tcyA*, *tcyB*, and *tcyC* genes (Table 2), presumably involved in L-cysteine transport, were detected only in ST123. Previous studies showed that *S. suis* strain 10 (ST1) is not auxotrophic for cysteine [42], and ST1 isolates lacked cysteine transporter homologs. The presence of this system in ST123 isolates therefore indicates lineage-specific differences in amino acid biosynthesis or acquisition. As L-cysteine is at low concentrations (~0.03 mM) in host fluids such as blood plasma, this transport may enhance bacterial survival during sepsis.

### Lineage-specific genes involved in regulation

Up to 23 genes encoding putative regulators were detected in ST1 genomes, including 9 linked to transcription, 5 to post-translational modification, 4 to stress response, 2 to quorum sensing, and 1 each to cell division, redox reactions, and signal transduction. The DeoR/GlpR-like regulator is placed on a metabolic island (Figure 1D). GlpR, a DeoR/LacI family repressor, controls glycerol metabolism by repressing *pflA1*, *pflB2*, and *fsaA* genes in the absence of glycerol [43], thus regulating expression of the metabolic island. GlpR has also been related to virulence, as it promotes resistance to oxidative stress and survival of *S. suis* strain SC070731 (serotype 2) in macrophages and mouse infection models [43]. Another ST1-specific regulator, RevS, is an orphan response regulator whose deletion reduces colonization of internal organs in piglets without affecting in vitro growth [44]. *revS* is also linked to highly virulent serotypes [45]. Additionally, *relE/B* encode a type II toxin–antitoxin system. RelE encodes a ribonuclease while RelB neutralizes its activity. These systems are required for plasmid maintenance and cell growth, and they are not considered classic regulators. However, work in *S. pneumoniae* showed that they repress their own operon [46], and enhance stress tolerance, antibiotic persistence, and biofilm survival in *Streptococcus mutants* [47].

In ST123 genomes, 13 putative regulatory genes were identified. Seven related to transcription, three to post-translational modification, two to DNA methylation, and one to epigenetic regulation. Among them, *licT* and an *araC-*-type regulator are located in the same metabolic island (Figure 1G. The LicT protein is a BglG-family transcription regulator that activates *bglP* and represses *bglA* in *S. mutans* [48], and regulates aesculin transport in response to glucose [48], suggesting independent control of both catalytic modules within the island. Another AraC-type regulator, SptRs in *S. sanguinis* represses genes related to biofilm formation, immune evasion, and oxidative stress tolerance [49]. MerR-type regulators, including *merR*, were identified adjacent to *murM* and *murN* in another metabolic island (Figure 1F). In *S. pneumoniae* and *Neisseria gonorrhoeae*, MerR proteins regulate *adhC* involved in H_2_O_2_ metabolism [50], mediate defense against carbonyl and nitrosative stress [51], and enhance oxidative stress survival in macrophages [52]. Altogether, differences in metabolic regulation between both STs may be coupled to different virulent profiles.

### Lineage-specific genes involved in virulence and defense

Virulence-associated genes in ST1 lineage included those related to capsule biosynthesis, adhesion, and immune evasion (Additional file 1). ST1 isolates used in this study produce capsule types 2, 1, 1/2, and 14. Serotype 2 capsule is composed of a tetrasaccharide repeat [β-D-Galactose-(1 → 4), β-D-Glucose-(1 → 3), β-D-Galactose-(1 → 4), and β-D-N-Acetylglucosamine] encoded by five core genes (*cps2K*, *neuB*/*cps2P*, *cps2Q*, *cps2R*, and *neuA*/*cps2S)*. Glycosyltransferases (Cps2E, Cps2K, Cps2Q, and Cps2R) assemble the polymer, while Wzx (*cps2E*) and Wzy (*cpsF*) mediates its export. Sialylation is carried out by the NeuC/NeuB/NeuA pathway. The serotype 2 capsule is a well-known virulence factor in *S. suis* [53] and its virulence is largely attributed to its sialic acid content [54]. Deletion of *neuB* yields uncapsulated strains with an attenuated virulence in animal models [55, 56]. However, STI isolates can also produce serotype 1 capsule, which lacks sialic acid but contains N-acetylgalactosaminuronic acid, indicating that other capsule traits or factors can compensate for sialic acid absence.

ST1 genomes contain a disrupted *hylA* gene (Figure 1D) divided into four fragments [57]. One, *hylS’,* encodes a putative secreted protein that interacts with component C3b, reducing its deposition on the bacterial surface and thereby enhancing immune evasion [57]. The gene encoding the major pilus subunit (*sbp2*) is also disrupted by nonsense mutations, resulting in two separate fragments. Yet, deletion of its 5´-fragment reduced virulence of *S. suis* strain P1/7 (serotype 2) in a zebrafish model and decreased adherence to Hep-2 cells [58]. Recombinant Sbp2 protein is immunogenic and protective in mice [59], although its proposed role as an adhesin is difficult to reconcile with the absence of secretion signals and anchoring motifs. In addition, ST1 genomes encode three sortases (*srtB*, *srtC*, and *srtD),* membrane transpeptidases that attach LPXTG- containing proteins to the cell wall. Sortases are not virulent factors themselves, but they process many cell walls associated virulence factors. SrtC participates in pilus assembly in *S. sanguinis* [60], *S. pneumoniae* [61], and *S*. *agalactiae* [62]; *srtC* mutant in the latter species significantly impaired adhesion, invasion, and virulence [61]. In *S. suis*, a *srtB* mutant resulted in highly attenuated invasive capacity in pigs, with no clinical signs of disease and no bacterial recovery from internal organs [63]. Regarding defense, ST1 isolates carry two genes encoding endonuclease subunit S proteins. One corresponds to a fragment of *hsdS* (SSU1271 in P1/7). In group A streptococci, *hsdS* and *hsdM* (methyltransferase) mark host DNA and cleave foreign sequences [64], reducing the incorporation of novel genetic elements. Moreover, in *S. suis, hsdS* inactivation reduced bacterial survival inside phagocytes, in blood, and under oxidative stress [65], suggesting a role in virulence.

ST123 isolates produce a serotype 9 capsule, a branched polysaccharide lacking sialic acid, with the repeating unit β-D-Glucopyranose(1 → 3) β-D-Galactofuranose-(1 → 3) β-D-Galactofuranose. The biosynthesis operon consists of five genes (*cps9E, cps9F*, *cps9G*, *cps9H*, and *cps2I*). Capsule production involves UDP-Galactofuranose synthesis by *glf*, transfer of sugar residues by Cps9 glycosyltransferases, polymerization and export by Wzx and Wzy and anchoring to the peptidoglycan. Unlike serotype 2, ST123 lacks sialic acid biosynthetic genes. Its absence in capsule 9 is expected to reduce resistance to complement and phagocytosis [66], but a serotype-switch experiment replacing capsule 2 with capsule 9 in strain P1/7 showed not significant change in adhesion, invasion, or macrophage resistance [67]. Beyond the capsule, ST123 genomes encode several hypothetical proteins with secretion signals that may contribute to virulence. Some lack LPXTG motifs, transmembrane domains, or lipoboxes, suggesting extracellular release. For example, gene UN722_00706 encodes a 216-amino-acid protein sharing 37% identity with the serum opacity factor of *Streptococcus porcinis,* which binds apolipoprotein A-I in high-density lipoproteins, reorganizing host lipids [68]. This process may help streptococci of ST123 to interfere with host lipid metabolism. Another example is gene UN722_01125, which encodes a large 979-amino-acid protein containing at its N-terminus a divergent LnlB-repeat domain originally described in *Listeria* virulence protein Internalin B [69]. This protein also harbors a NosD domain, characteristic of copper-binding/export proteins involved in maturation of copper-dependent enzymes and stress resistance. In addition, serum opacity factors often contain fibronectin-binding domains, potentially contributing to bacterial adhesion. Gene UN722_01790 encodes a 126-amino acid secreted protein with consecutive C-terminal tryptophan residues associated with surface and lipid interactions probably contributing to adhesion [70]. Other predicted surface proteins contain LPXTG motifs, such as UN722_01091, encoding a large 897-amino-acids secreted protein with coiled-coil regions typical of adhesins. Although experimental validation is pending, these proteins are strong candidates for novel virulence factors in ST123 isolates. Regarding defense mechanisms, ST123 genomes contain three type II restriction endonucleases, two endonuclease subunit S proteins, one bleomycin resistance protein, and a truncated *hsdS* ortholog (NCTC10234_00670). These differences in the repertoire of defense-related genes may explain the evolutionary rate of each ST lineage.

### Analysis of virulence in mice infection models

**Figure 2:**
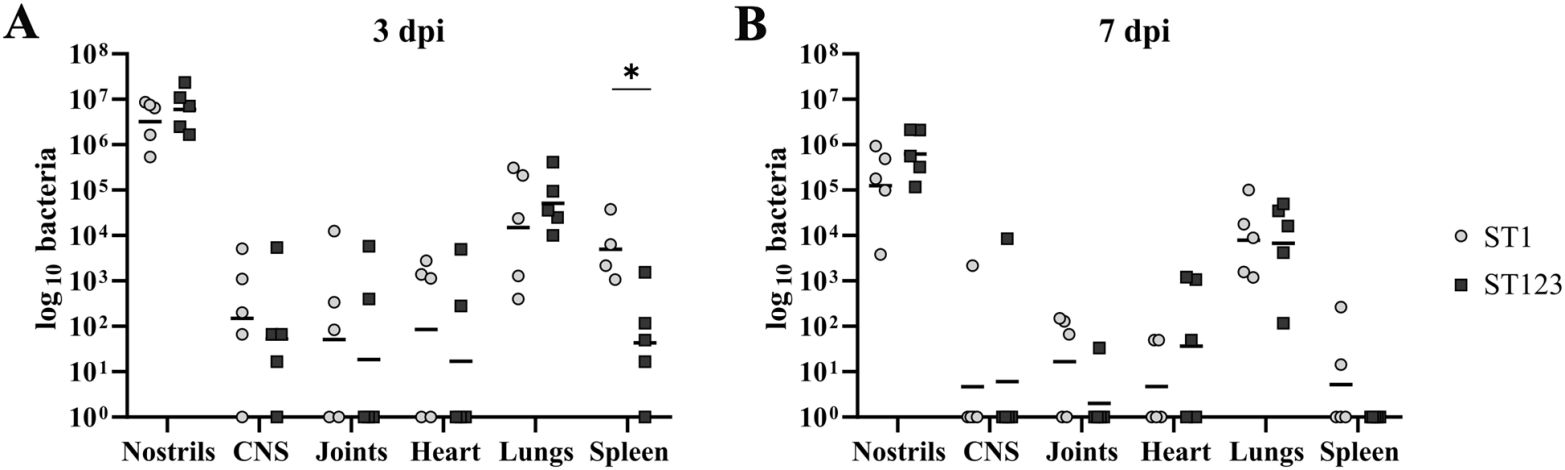
**Infection assays using an intranasal murine chronic infection model.** Colony-Forming Units (CFU) of *S. suis* isolates from ST1 (Ss_02, Ss_22, Ss_31, Ss_80, and Ss_151) and ST123 (Ss_84, Ss_100, Ss_106, Ss_110, and Ss_156) were quantified in various organs at **A** 3 days post-infection (dpi), and **B** 7 dpi. Each data point represents the mean CFU count per organ from 6 mice, with 5 biological isolates tested per ST. Statistical significance (*p* < 0.05) is indicated by an asterisk.

Our genetic analysis evidenced differences in the repertoire of virulence factors between the two lineages. To compare their virulence potential, five ST1 isolates (Ss_02, Ss_22, Ss_31, Ss_80, and Ss_151) and five ST123 isolates (Ss_84, Ss_100, Ss_106, Ss_110, and Ss_156) from different geographic origins were analyzed using a murine intranasal infection model. Bacterial loads were quantified in the nostrils, lungs, and internal organs by CFU enumeration at 3- and 7-days post-infection (dpi) (data collected in Additional file 2). No symptoms of disease were detected in any mice before sacrifice. At 3 dpi, all ST1 isolates were recovered from the nostrils, with bacterial loads ranging from 8 × 10^5^ to 2 × 10^7^ CFU/g (Figure 2A), indicating successful colonization. They were also detected in the lungs (average 1.09 × 10^3^ CFU/g). Additionally, all ST1 isolates were recovered from the spleen (average 9.43 × 10^3^ CFU/g), and three of the five strains were detected in the joints (average 2.57 × 10^3^ CFU/g) and heart (average 1.06 × 10^3^ CFU/g), indicative of systemic dissemination. Four of the five ST1 isolates were also detected in the brain (average 1.31 × 10^3^ CFU/g), demonstrating their ability to invade the central nervous system (CNS). The detection of ST1 isolates in the CNS is in agreement with previous reports linking this ST to neuroinvasive disease in pigs in the USA [71]. By 7 dpi, ST1 isolates persisted in all analyzed organs, mainly in the lungs, but a ~100-fold load reduction was detected in nostrils, brain, joints, heart, and spleen (Figure 2B), demonstrating clearance mechanisms.

ST123 isolates showed similar but slightly higher colonization loads in the nostrils and lungs at 3 dpi compared with ST1 isolates (Figure 2A), with average loads of 9.15 × 10^6^ CFU/g, and 1.16 × 10^5^ CFU/g, respectively. Systemic dissemination was also evident for ST123 isolates, with considerable bacterial loads in two isolates in the joints (average 1.23 × 10^3^ CFU/g), four in the CNS (average 1.11 × 10^3^ CFU/g), two in the heart (average 1.06 × 10^3^ CFU/g), and four in the spleen (average 3.47 × 10^2^ CFU/g). Remarkably, bacterial counts in the spleen were significantly lower in ST123 compared with ST1 isolates. Although not statistically significant due to interstrain variation, lung colonization tended to be higher in ST123 than in ST1 isolates, in agreement with the slightly higher bacterial loads observed in the nostrils. At 7 dpi, bacterial loads in the nostrils decreased approximately tenfold but remained higher than those observed for ST1 isolates (Figure 2B). Lung bacterial loads also decreased by about tenfold. ST123 isolates showed reduced persistence in internal organs after 7 dpi, as none were recovered from the spleen, and only one isolate was detected in the joints and CNS. In contrast, bacterial loads in the heart increased compared with 3 dpi and were higher, though not significantly, than those observed for ST1 isolates. These findings suggest that ST123 isolates may be better adapted than ST1 for colonization of the nasopharyngeal and heart tissues, where ST1 isolates exhibit higher colonization of the spleen, joints, and CNS.

### Interaction with macrophages

**Figure 3:**
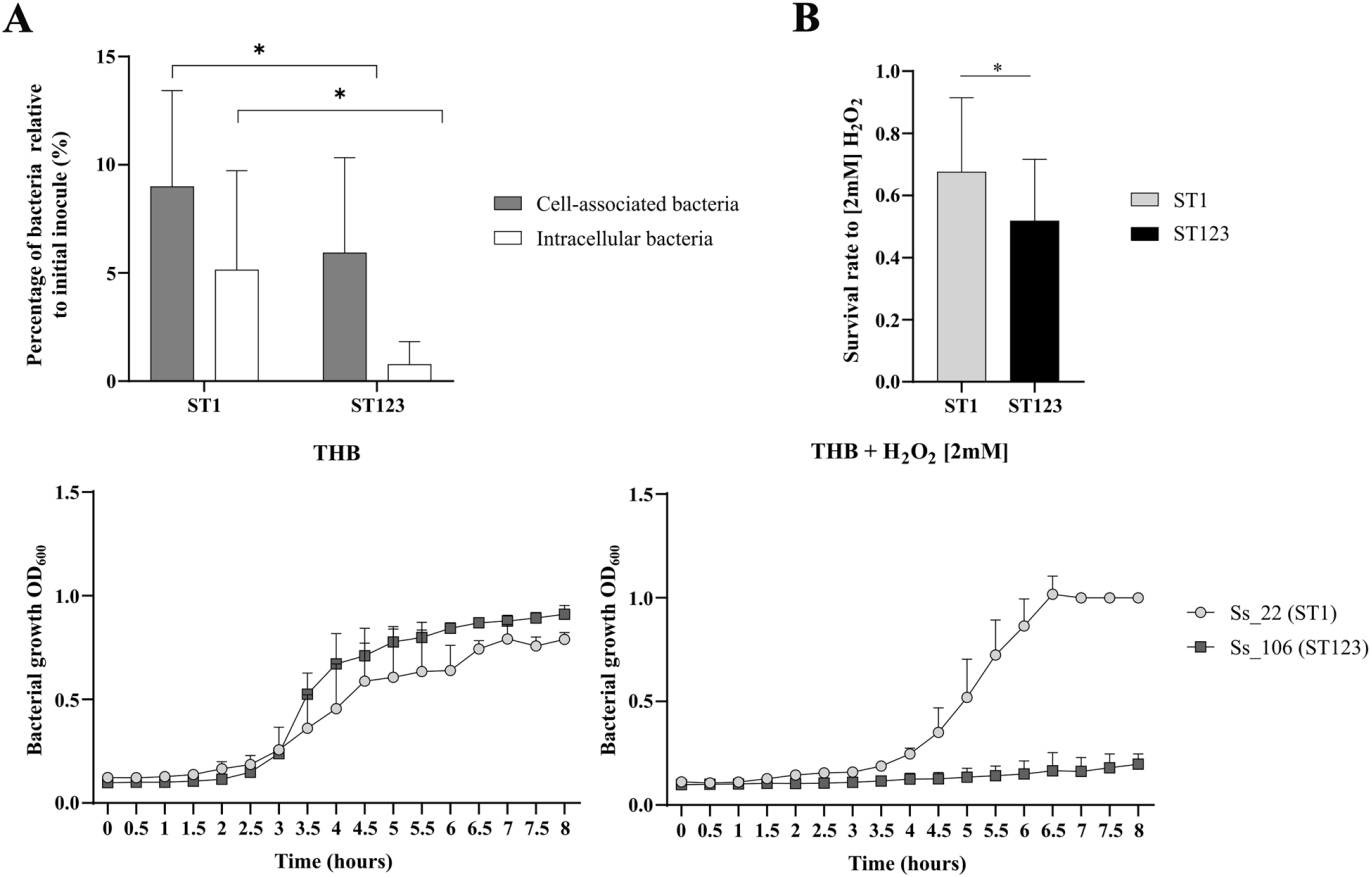
**Interaction of bacteria with macrophages and tolerance to oxidative stress induced by H**_**2**_**O**_**2**_. **A** Percentage of cell-associated and intracellular bacteria from ST1 (Ss_02, Ss_22, Ss_31, Ss_80, and Ss_151) and ST123 (Ss_84, Ss_100, Ss_106, Ss_110, and Ss_156) isolates in activated J774A.1 macrophages relative to the original bacterial inoculum and to the cell-associated bacteria, respectively. Average values from at least four assays in five isolates per ST are shown. **B** Average survival rate of the same five isolates per ST, calculated by comparing the area under the growth curve after 8 h of incubation in THB alone and in THB supplemented with 2 mM of H_2_O_2_. Bacterial growth curves of representative isolates Ss_22 (ST1) and Ss_106 (ST123) in both media (THB and THB with 2 mM of H_2_O_2_) are shown in the lower-left and lower-right panels. Statistically significant differences between groups (*p* < 0.05) are indicated by an asterisk.

Phagocytosis is a critical host defense mechanism against *S. suis*. To assess the ability of ST1 and ST123 isolates to evade phagocytic clearance, we evaluated their adherence to and uptake by murine macrophages. Macrophages were stimulated with LPS and infected with bacterial isolates at a multiplicity of infection of 100. At 1.5 h post-infection, cell-associated and intracellular bacteria were quantified by CFU counting. The results are shown in Figure 3A. ST1 isolates exhibited an average of 2.79 × 10^6^ macrophage-associated bacteria, representing approximately 9% of the total inoculum. Among these, 1.39 × 10^5^ CFU were detected intracellularly, corresponding to 5% of the total cell-associated bacteria. Conversely, ST123 isolates associated less efficiently with macrophages and showed reduced intracellular survival compared with ST1. An average of 9.62 × 10^5^ CFU were associated with macrophages, corresponding to 5.9% of the inoculated bacteria. Indeed, 2.1 × 10^4^ CFU were found intracellularly, corresponding to 0.8% of the total cell-associated bacteria. Together, our data indicate that ST123 isolates evade interaction with macrophages more effectively than ST1 isolates, but are more susceptible to phagocytic killing, evidencing differences in host–pathogen interactions between both STs. A previous study showed that a capsule 9 strain was internalized more efficiently than two strains of serotypes 2 and 14 [66]. These differences were caused by the capsule, as depletion of capsule production in all strains enhanced the number of intracellular bacteria at similar levels. Thus, the differences observed between both STs in our work may not be explained by differences in capsule production. However, it is important to note that the authors only tested one strain of each serotype.

### Bacterial growth under stress conditions

The low intracellular recovery of ST123 bacteria in macrophage assays may reflect a reduced ability to withstand host antimicrobial mechanisms such as oxidative stress. When macrophages phagocytose bacteria, they activate the NADPH oxidase enzyme complex, generating superoxide radicals in the phagosome. These radicals are subsequently converted to hydrogen peroxide by superoxide dismutase. The H_2_O_2_ generates reactive oxygen species that damages bacterial components and structures through oxidative stress. To explore the oxidative stress resistance of the five isolates of ST1 (Ss_02, Ss_22, Ss_31, Ss_80, and Ss_151) and five isolates of ST123 (Ss_84, Ss_100, Ss_106, Ss_110, and Ss_156), we compared their growth in THB supplemented with 2 mM H_2_O_2_ for 8 h. Growth was quantified by calculating the area under the growth curve from three independent experiments. ST1 isolates exhibited robust growth in the presence of H_2_O_2_, whereas ST123 isolates showed a significant reduction in growth under oxidative conditions (Figure 3B). Together, these data indicate that ST123 isolates have reduced tolerance to oxidative stress compared with ST1 isolates, which may suggest a reduced capacity to evade phagocytic killing.

### Analysis of β-lactam resistance generation

**Figure 4:**
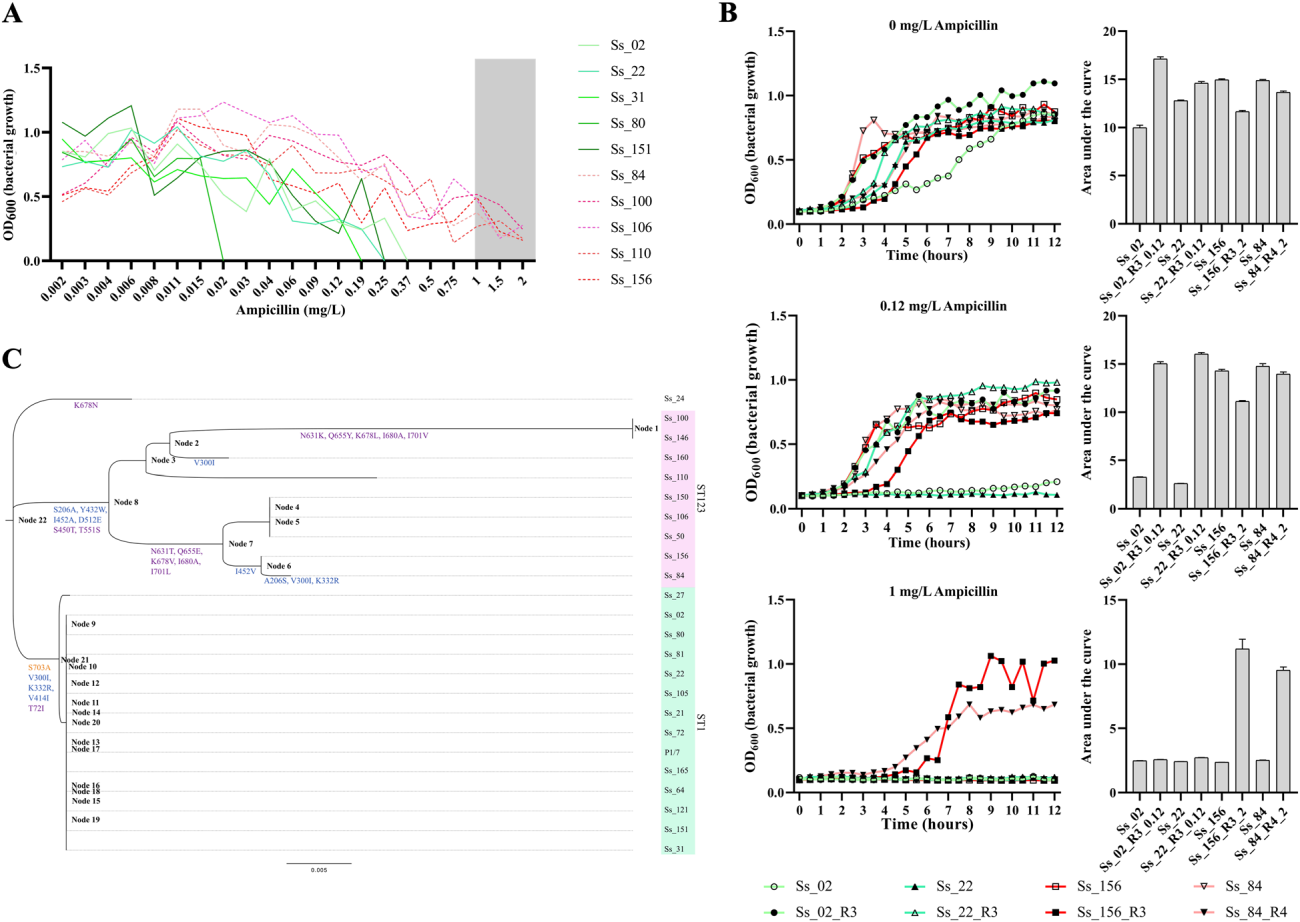
**Generation of ampicillin resistance in ST1 and ST123 isolates**. **A** Serial passaging of *S. suis* isolates of ST1 (Ss_02, Ss_22, Ss_31, Ss_80, and Ss_151) and ST123 (Ss_84, Ss_100, Ss_106, Ss_110, and Ss_156) in THB with increasing concentrations of ampicillin. Bacterial growth was measured at OD_600_. The shaded area represents the range corresponding to intermediate ampicillin resistance. ST1 isolates are shown as solid green lines, while ST123 isolates are shown as dotted red lines. **B** Growth curves of spontaneous ampicillin-resistant clones (black-filled symbols) obtained by serial subculturing of *S. suis* in increasing concentrations of ampicillin, compared with their corresponding wild-type strains (empty symbols) at 0 mg/L and 1 mg/L ampicillin. The area under the curve from three independent experiments is shown on the right panel. **C** Ancestral reconstruction of β-lactam resistance variants based on amino acid sequences of PBP1A, PBP2B, and PBP2X proteins from ST1 and ST123 isolates through their phylogenetic history. The isolate Ss_24 of ST949 was used as an outgroup. Mutations in PBP1A, PBP2B, and PBP2X are shown in orange, blue, and purple, respectively. In all panels, ST1 isolates are shown in red hues, and ST123 isolates in green hues.

In previous studies, we detected that invasive ST123 isolates had higher β-lactam resistance than ST1 isolates [12, 72]. To investigate whether ST123 isolates possess an enhanced capacity to develop β-lactam resistance, we evaluated the spontaneous acquisition of resistance to ampicillin. ST1 and ST123 isolates, which were sensitive to ampicillin (Table 1), were exposed to serially increasing ampicillin concentrations and growth was assessed by final OD_600_ measurements at each concentration. ST1 isolates showed robust growth at ampicillin concentrations between 0.002 and 0.004 mg/L, but at concentrations above 0.006 mg/L their growth yield reduced drastically (Figure 4A). The isolate Ss_80 from the ST1 lineage only grew at concentrations up to 0.015 mg/l, while the remaining four showed limited growth between 0.12 and 0.19 mg/L of ampicillin. In contrast, all ST123 isolates grew at concentrations up to 2 mg/L of ampicillin, although their growth yield was reduced above 0.19 mg/L (Figure 4A).

To further assess resistance acquisition, colonies of isolates Ss_02 and Ss_22 (ST1) were isolated during the course of the experiment from liquid cultures at 0.12 mg/L ampicillin, and isolates Ss_84 and Ss_156 (ST123) at 0.5 and 2 mg/L by placing them onto THA plates. The isolated clones were designated as Ss_02_R3_0.12, Ss_22_R3_0.12, Ss_84_R4_0.5, Ss_84_R3_2, Ss_156_R3_0.5, and Ss_156_R1_2 (Table 1). Growth curves were performed for the clones in THB supplemented with or without ampicillin, and the results evidenced enhanced resistance to ampicillin (Figure 4B). Their clinical resistance to ampicillin was determined by MIC, resulting in 0.12 mg/L for Ss_02_R3_0.12 and Ss_22_R3_0.12, 0.5 mg/L for Ss_84_R4_0.5 and Ss_156_R3_0.5, 2 mg/L for Ss_156_R3_2, and 4 mg/L for Ss_84_R4_2 (Table 1). Thus, some ST123 clones demonstrated a significantly higher tendency to develop resistance to ampicillin compared with ST1 isolates.

β-lactam resistance is mainly caused by mutations in genes encoding penicillin-binding proteins (PBP1a, PBP2b, and PBP2x), mainly in the *pbp2x* gene [73]. We sequenced the *pbp2x* gene in all ampicillin-resistant clones, and the sequences were compared with those of their sensitive parents. For all ST1 resistant clones isolated at 0.12 mg/L ampicillin only one mutation per clone was detected and it was located within the transpeptidase domain of PBP2X (residues 292–592) (Additional file 3A). Regarding ST123 isolates, all ampicillin-resistant clones of Ss_156 showed no mutations in *pbp2x* as compared with the wild-type (data not shown), while all ampicillin-resistant clones of Ss_84 showed several mutations (Additional file 3B). The absence of new substitutions in Ss_156 clones may be explained by the fact that PBP2X protein of the wild-type strain already contained several substitutions associated with ampicillin resistance [12], and these clones may therefore rely on alternative mechanisms to achieve increased resistance. Three of the mutations were located within the transpeptidase domain. Interestingly, the S556G mutation in PBP2X was detected in all five clones resistant to 2 mg/L ampicillin analyzed in the present study (Ss_84_R1_2, Ss_84_R2_2, Ss_84_R3_2, Ss_84_R4_2, and Ss_84_R5_2) (Additional file 3B). and was previously detected in β-lactam resistant *S. suis* isolates [73, 74] and in ST123 strain Ss_160 from our collection. Notably, it was detected in 71% of the *S. suis* collection of diseased pigs in Thailand between 2018 and 2020 [74]. Thus, certain mutations appear to become fixed and contribute to the further development of antibiotic resistance. We also investigated whether similar resistance patterns occurred in natural environments by analyzing adaptive sequence changes in PBP1A, PBP2B, and PBP2X, using genomic sequences from nine ST123 isolates that showed MIC values to ampicillin of < 0.06 mg/L (*n* = 1), 0.12 mg/L (*n* = 4), 0.25 mg/L (*n* = 2), and 0.5 mg/L (*n* = 2). As controls, we included 13 ST1 isolates and the ST949 isolate Ss_24 [3] as an outgroup. We focused on mutations previously associated with β-lactam resistance [12]. The results are shown in Figure 4C. All ST123 sequences evolved from a most recent common ancestral sequence (corresponding to node 22) and shared relevant mutations (S206A, Y432W, I452A, and D512E in PBP2B and S450T and T551S in PBP2X). In contrast, ST1 isolates shared identical PBP1A, PBP2B, and PBP2X sequences, except for isolate Ss_27 (Figure 4C). These findings indicate that ST1 and ST123 evolved by fixing different resistance-associated amino acids changes. In general, ST123 isolates exhibited higher sequence variability and a trend toward an increased rate of molecular evolution in these proteins compared with ST1. These findings agree with our culture assays under laboratory conditions where antibiotic resistance evolved based on fixed mutations, which is supported by a higher mutation rate found in ST123 lineage. A recent study in *S. pneumoniae* indicated spontaneous mutations in the *pbp2x* gene after progressively increased penicillin concentrations or through interspecies recombination [27]. The mutants generated through recombination showed no loss of virulence in mice, while those arising from spontaneous mutations were avirulent [27]. This could be explained considering that mutations in PBPs often lead to reduced growth. ST1 isolates are considered hypervirulent but are often very sensitive to β-lactams, whereas ST123 isolates, although highly virulent, show high levels of penicillin resistance.

### Biofilm formation and its tolerance to antibiotics

**Figure 5:**
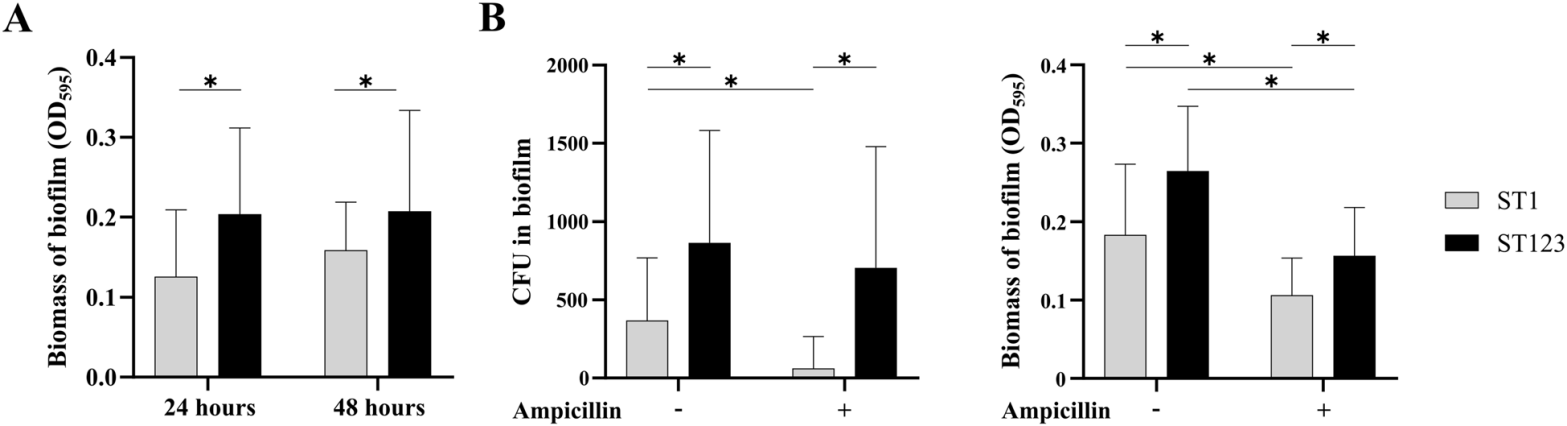
**Biofilm formation and antibiotic tolerance in ST1 and ST123 isolates.** **A** Biofilm formation quantified after 24 and 48 h of incubation of ST1 (Ss_02, Ss_22, Ss_31, Ss_80, and Ss_151) and ST123 (Ss_84, Ss_100, Ss_106, Ss_110, and Ss_156) isolates. **B** Tolerance to ampicillin under biofilm conditions, evaluated by quantifying colony-forming units (CFU) from five ST1 and five ST123 isolates in 24 h-old biofilms incubated for an additional 24 h in THB medium supplemented with ampicillin at MIC concentrations (+) or in nonsupplemented THB (−) (left panel). The biofilm biomass was also quantified after ampicillin treatment (right panel). Statistically significant differences (*p* < 0.05) are indicated with an asterisk.

Biofilm formation is relevant to host colonization and endocarditis and contributes to increased antibiotic tolerance and resistance [75, 76]. Several lineage-specific genes as the *cps* genes, adhesion genes (*sbp2*, *spaA*, *sfp1*, and *strC* in ST1; and *comGE*, UN722_01790 in ST123), and regulatory genes (U0698_00077 (LacI regulator) in ST1, and UN722_00876 (AraC regulator) UN722_01607 (MerR regulator), UN722_01088 (MutR regulator in ST132) were potentially linked to biofilm formation. Because differences were observed between both lineages regarding colonization of nostrils and heart tissues, we hypothesized that ST123 might have an increased capacity for biofilm production. To investigate this, we compared the biofilm-formation capacity of ST1 and ST123 after 24 and 48 h of incubation. At 24 h, ST1 isolates produced significantly less biofilm biomass than ST123 isolates, specifically 1.62-fold less (Figure 5A). Although biofilm formation by ST1 increased after 48 h, it remained significantly lower than that of ST123 (Figure 5A). These findings confirm that ST123 isolates indeed have a greater intrinsic capacity for biofilm formation compared with ST1. This result is in agreement with a recent study showing that invasive isolates of serotype 2 (linked to the ST1 lineage) produced less biofilm biomass than those of serotype 9 (linked to the ST123 lineage) [77].

The enhanced biofilm production observed in ST123 may contribute to its persistence in clinical settings, particularly in environments where β-lactams are routinely used. We then evaluated the antibiotic tolerance of these biofilms. The 24-h biofilms were treated with ampicillin at the MIC concentration, followed by an additional 24-h incubation. In ST1 biofilms, an average of 367 CFU were recovered from untreated biofilms, while only 61 CFU were recovered after ampicillin treatment (Figure 5B), corresponding to a survival rate of 16%. In contrast, ST123 biofilms yielded an average of 865 CFU in untreated controls and 705 CFU after ampicillin exposure, corresponding to a survival rate of 81.5%, which was significantly higher than that observed in ST1 biofilms. Accordingly, ampicillin reduced biofilm biomass in both STs, but the remaining biomass was still higher in ST123 than in ST1 (Figure 5B). These results suggest that the enhanced biofilm-forming capacity or the structural properties of ST123 biofilms may contribute to their ability to withstand antibiotic treatment in the host. Indeed, biofilm formation is a well-known mechanism of antibiotic tolerance in *S. suis* [78]. For example, a previous report showed that biofilm cells may require up to 16 times the concentration of penicillin G needed to inhibit planktonic cells [79]. However, in other streptococcal species such as *S. agalactiae,* biofilm formation has not been linked to β-lactam resistance [80].

In summary, this study highlights key genotypic differences between the two dominant invasive *S. suis* lineages circulating in Spain, including high sequence variability, divergence in core genes content, and distinct sets of lineage-specific genes involved in metabolism, nutrition, regulation, and virulence. Phenotypic assays of representative isolates from both STs revealed that ST123 isolates exhibit an enhanced capacity for β-lactam resistance, increased biofilm-forming capacity, reduced interaction with macrophages, and improved adaptation to nasal colonization compared with ST1 isolates. In contrast, ST1 isolates were more invasive in animal models, showed higher resistance to macrophage killing and oxidative stress, but remained more susceptible to β-lactams. These findings suggest that the two lineages have adopted distinct evolutionary strategies to promote survival and persistence within host. The rapid emergence of *S. suis* ST123 in Spain and its recent expansion to Italy underscore the need for genomic surveillance and monitoring antimicrobial resistance trends in this emerging lineage.

## Supplementary Information

**Additional file 1.** **Lineage-specific genes of ST1 (sheet 1) and ST123 (sheet 2).**

**Additional file 2.** **Bacterial survival during an intranasal murine chronic infection assay.** Colony Forming Units (CFU) of *S. suis* isolates from ST1 (Ss_02, Ss_22, Ss_31, Ss_80, and Ss_151) and ST123 (Ss_84, Ss_100, Ss_106, Ss_110, and Ss_156) are indicated individually per organ for 3 and 7 days post-infection (dpi).

**Additional file 3.** **Multiple alignment of PBP2X amino acid sequences in spontaneous ampicillin resistant clones.** Dots indicate identity with the sequence of the sensitive parental strain. (**A**) ST1 isolates Ss_02 and Ss_22, resistant to 0.12 mg/L of ampicillin, aligned against the sequence of the parental isolate Ss_22. (**B**) ST123 isolate Ss_84, with clones resistant to 0.5 and 2 mg/L ampicillin. Only sequences containing mutations are shown. The transpeptidase domain is highlighted in light orange. Amino acidic substitutions are marked in blue, and their position are indicated.

## Data Availability

All data generated in this work are available as main figures or in supplementary files. The genomes used in this work are publicly available on line under the NCBI numbers: Ss_02 (NZ_JAWWZM010000000), Ss_21 (NZ_CP139881), Ss_22 (NZ_CP139880), Ss_72 (NZ_JAXKWL000000000), Ss_80 (NZ_JAWWZJ010000000), Ss_121 (NZ_JAWWZF010000000), Ss_84 (NZ_JAXKWK010000000), Ss_100 (NZ_JAWWZG010000000), Ss_106 (NZ_JAXKWJ010000000), and Ss_156 (NZ_JAWWZE010000000), The rest of the genomes are under the biosample number: Ss_27 (SAMN47684797), Ss_31 (SAMN47684798), Ss_64 (SAMN47684801), Ss_81 (SAMN47684802), Ss_105 (SAMN47684803), Ss_151 (SAMN47684807), Ss_165 (SAMN47684809), Ss_50 (SAMN47684799), Ss_110 (SAMN47684804), Ss_146 (SAMN47684805), Ss_150 (SAMN47684806), and Ss_160 (SAMN47684808). All recollected under the BioProjects with references PRJNA1037519, PRJNA1037519 and PRJNA1037513 in the NBCI database and, they are also available on zenodo (10.5281/zenodo.19581781).
