# Supplementary figures and images for: Clinically relevant genomic and phenotypic differences in virulence, antimicrobial resistance, and biofilm-associated tolerance between Streptococcus suis lineages ST1 and ST123
Source: Vet Res. 2026 Jun 12;57:105. doi: 10.1186/s13567-026-01782-2 (PMC13262394; doi:10.1186/s13567-026-01782-2)

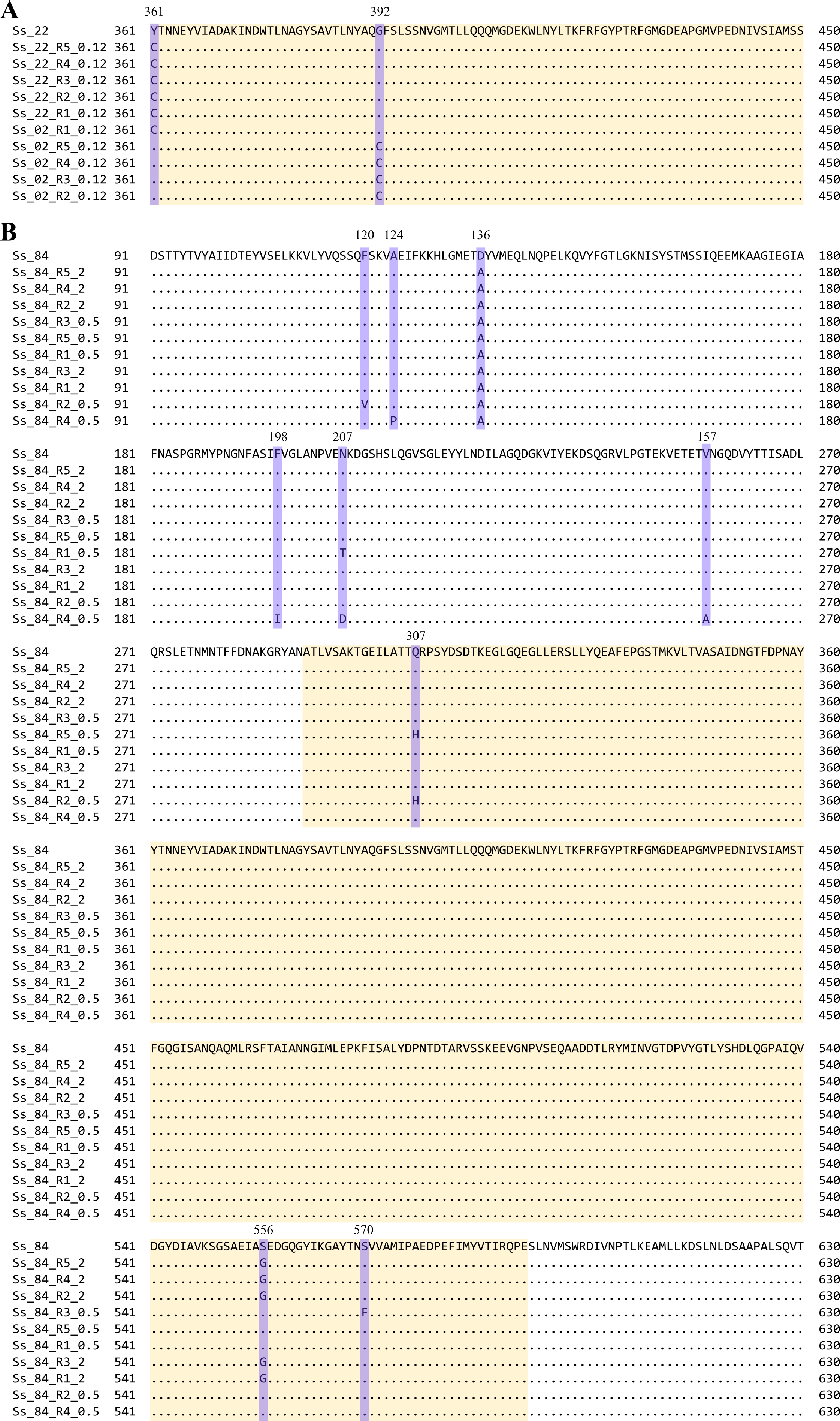

Supplement: Supplementary file 3 — Additional file 3. Multiple alignment of PBP2X amino acid sequences in spontaneous ampicillin resistant clones. Dots indicate identity with the sequence of the sensitive parental strain. (A) ST1 isolates Ss_02 and Ss_22, resistant to 0.12 mg/L of ampicillin, aligned against the sequence of the parental isolate Ss_22. (B) ST123 isolate Ss_84, with clones resistant to 0.5 and 2 mg/L ampicillin. Only sequences containing mutations are shown. The transpeptidase domain is highlighted in light orange. Amino acidic substitutions are marked in blue, and their position are indicated. [file 13567_2026_1782_MOESM3_ESM.tiff]
